# On the limits of language influences on numerical cognition – no inversion effects in three-digit number magnitude processing in adults

**DOI:** 10.3389/fpsyg.2015.01216

**Published:** 2015-08-12

**Authors:** Julia Bahnmueller, Korbinian Moeller, Anne Mann, Hans-Christoph Nuerk

**Affiliations:** ^1^Knowledge Media Research CenterTuebingen, Germany; ^2^Department of Psychology, Eberhard Karls UniversityTuebingen, Germany; ^3^LEAD Graduate School, Eberhard Karls UniversityTuebingen, Germany

**Keywords:** multi-digit number comparison, three-digit numbers, compatibility effects, language-moderated effects, developmental changes

## Abstract

The inversion of number words influences numerical cognition even in seemingly non-verbal tasks, such as Arabic number comparison. However, it is an open question whether inversion of decades and units also influences number processing beyond the two-digit number range. The current study addresses this question by investigating compatibility effects in both German- (a language with inverted) and English-speaking (a language with non-inverted number words) university students (mean age 22 years) in a three-digit number comparison task. We observed reliable hundred-decade as well as hundred-unit compatibility effects for three-digit number comparison. This indicates that, comparable two-digit numbers, three-digit numbers are processed in a parallel decomposed fashion. However, in contrast to previous results on two-digit numbers as well as on children’s processing of three-digit numbers, no reliable modulation of these compatibility effects through language was observed in adults. The present data indicate that inversion-related differences in multi-digit number processing are limited. They seem to be restricted to the number range involving those digits being inverted (i.e., tens and units in two-digit numbers) but do not generalize to neighboring digits. Possible reasons for this lack of generalization are discussed.

## Introduction

Everyday life usually involves processing of multi-digit numbers. Nevertheless, much of the research in numerical cognition has been devoted to single-digit number processing. However, findings from single-digit number processing may not simply be transferred to multi-digit number processing (Nuerk et al., 2011). Indeed, specific processes and representations (e.g., base-10 place-value representation, the carry-process in addition) are exclusive to multi-digit number processing (Nuerk et al., 2015). Importantly, such multi-digit number representations are not only of academic interest but seem of particular relevance for numerical development. Moeller et al. (2011) showed that the mastery of the place-value structure of the Arabic number system in first grade predicted later calculation performance. In the following, we will first describe specificities of multi-digit numbers before discussing language influences on multi-digit number processing essential for the current study.

The majority of studies investigating multi-digit number processing focused on two-digit integer numbers (Nuerk et al., 2011, 2015 for overviews). Even though earlier studies concluded on holistic processing of two-digit numbers as integrated entities (e.g., Dehaene et al., 1990; see also Zhang and Wang, 2005; Ganor-Stern et al., 2009) there is accumulating evidence suggesting two-digit numbers to be processed in a decomposed manner (i.e., separated into tens and units, e.g., Nuerk et al., 2001; Ganor-Stern et al., 2007; Kallai and Tzelgov, 2012; Macizo and Herrera, 2013; Moeller et al., 2013).

However, for numbers beyond the two-digit number range empirical evidence is sparser and in contrast to what has been observed for two-digit numbers, recent studies suggest that higher multi-digit numbers are processed in a combined parallel-sequential manner. In an eye-tracking study, Meyerhoff et al. (2012) found that for four- to six-digit numbers processing of the constituting digits becomes less parallel and more sequentially clustered (see also Poltrock and Schwartz, 1984). On the other hand, Korvorst and Damian (2008) observed that three-digit numbers are primarily processed in parallel but with a left-to-right gradient reflecting the relevance of hundreds, tens, and units. Finally, Mann et al. (2012) showed that, for three-digit numbers, parallel decomposed processing developed later and in a less consistent way as compared to two-digit numbers. Taken together, this indicates that results from two-digit numbers cannot simply be transferred to higher multi-digit numbers.

It has long been reported that greater transparency of the number word system facilitates number processing and arithmetic performance at virtually all stages of development (e.g., Miura et al., 1988; Fuson and Kwon, 1991; Miura and Okamoto, 2003; Dowker and Lloyd, 2005; Dowker et al., 2008; Krinzinger et al., 2011). Noteworthy, number word structure influences number processing in verbal but also in other numerical tasks that do not rely on a verbal processing component explicitly. As regards verbal numerical tasks, specific transcoding errors were observed depending on specific characteristics of certain number word systems (e.g., effects of the base-20 system in the French number word system, Seron and Fayol, 1994; see also Colomé, À et al., 2010 for base-20 system effects in Basque). More specifically related to the current study are transcoding errors due to the inversion property of some languages. In German, Dutch, Arabic but also other languages the order of tens and units in number words corresponding to two-digit numbers is inverted as compared to the Arabic digital notation (e.g., 27 ↔ “siebenundzwanzig,” i.e., “seven-and-twenty”). As a consequence, children speaking a language with inversion commit specific inversion related errors in transcoding (i.e., writing down 72 when dictated “seven-and-twenty”). For German-speaking first graders it has been shown that almost 50% of errors committed are inversion related (Zuber et al., 2009). No such errors are reported for languages without inversion (e.g., Italian, cf. Power and Dal Martello, 1990, 1997). In a recent study in Czech, where both an inverted and a regular number word system exist for two-digit number words, revealed the same detrimental effects on transcoding even in a within-participant design in first-grade children (Pixner et al., 2011b; see also Imbo et al., 2014, for cross-linguistic effect within the same nation). However, number word influences on multi-digit number processing also extend to other numerical tasks such as symbolic magnitude comparison (Nuerk et al., 2005; Pixner et al., 2011a), number line estimation (Helmreich et al., 2011) or mental addition (Göbel et al., 2013).

The effect of interest, with which language influences can be studied in multi-digit number comparison, is the unit-decade compatibility effect. In unit-decade compatible number pairs separate comparisons of tens and units lead to the same decision (e.g., 42_57; 4 < 5 and 2 < 7) whereas in unit-decade incompatible number pairs separate comparisons of tens and units lead to opposing decisions (47_62, 4 < 6, but 7 > 2). Usually, unit-decade compatible pairs are responded to faster and less error prone than incompatible number pairs (e.g., Nuerk et al., 2001; Ganor-Stern et al., 2007, 2009; Moeller et al., 2009; Macizo et al., 2010; Macizo and Herrera, 2013; see Nuerk et al., 2011, 2015, for reviews). Importantly, this effect was shown to be modulated by the numerical distances between corresponding digit positions. Nuerk et al. (2001, see also Nuerk et al., 2004 for children data) found, for example, that the unit-decade compatibility effect was more pronounced when the distance between the unit digits of the two numbers of a number pair was large. This influence of the respective distances indicates that the unit-decade compatibility effect is not an attentional congruity effect or a categorical response conflict but is indeed driven by the separate processing of the numerical magnitudes of the constituent digits of a multi-digit number.

Furthermore, language and namely, inversion of number words, has been shown to influence the compatibility effects in different studies. For two-digit number pairs, it has been shown that the unit-decade compatibility effect is more pronounced for languages with number word inversion (Nuerk et al., 2005, see also Pixner et al., 2011a for children data), at least when numbers are read from left-to-right (Moeller et al., 2015). It has been argued that number word inversion influences the comparison process as the unit digit being named first in the respective number words (erroneously) implies a higher importance and activation of the unit digit, although it is actually irrelevant for the decision. The higher activation of unit digits elevates the compatibility effect, because it is actually a unit interference effect, where the automatic activation of irrelevant unit comparisons cannot be completely suppressed thus hindering or prolonging responses in incompatible trials.

**FIGURE 1:**
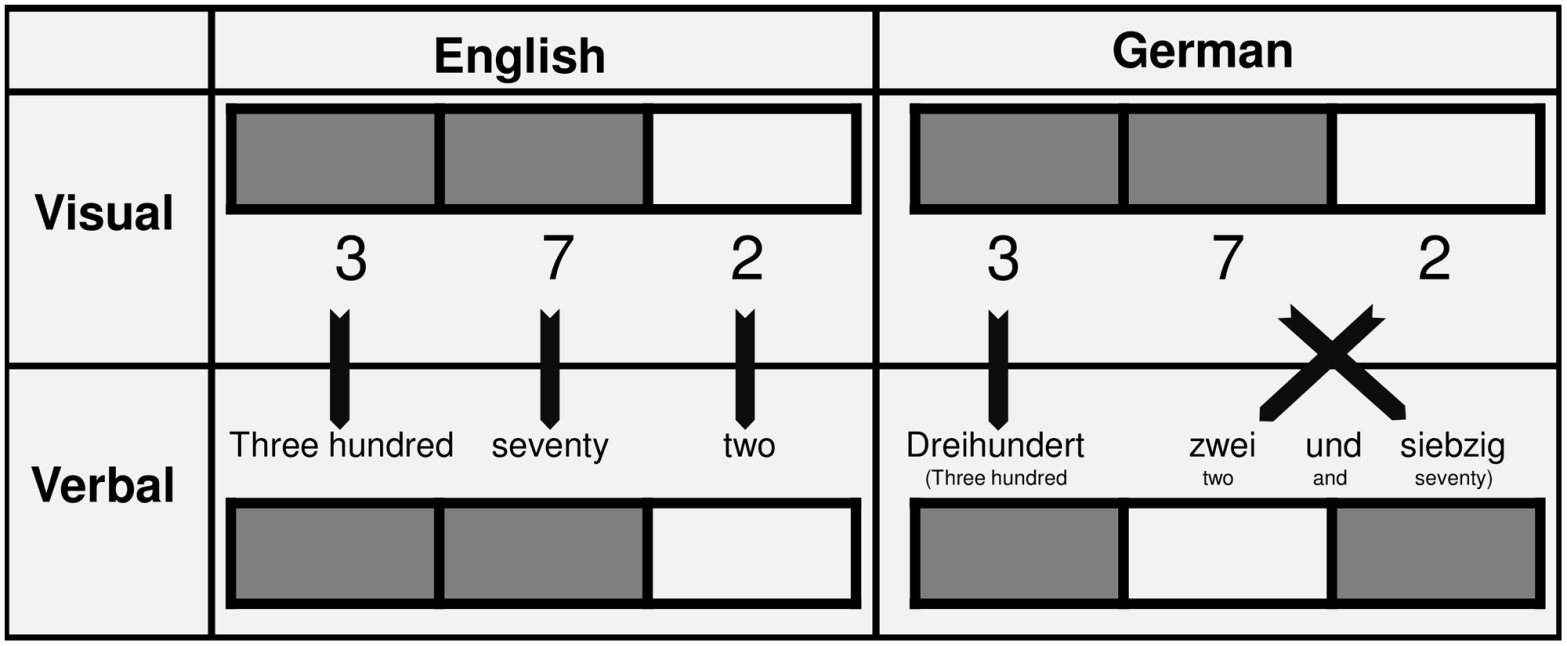
**Visual and verbal neighborhood**. If language influences are present, verbally neighboring digits should interfere with the comparison process. In case of a language without inverted number words, the interference due to the decade digits should be most pronounced. In contrast, for a language with inversion, the interference due to the unit digits should be more pronounced as compared to the decade digits.

In the current study we were interested in whether and – if so – how three-digit number comparison is influenced by inversion. In languages with non-inverted number words, the order of digits in a three-digit number is the same as the order of constituents of the corresponding number word (e.g., in English: 372 ↔ three-hundred-and-seventy-two). Contrarily, this is not the case in languages with inverted number words (e.g., in German: 372 ↔ three-hundred-two-and-seventy; see **Figure 1**, for illustration). Similar to two-digit numbers, the unit digit is named before the tens digit following the hundreds digit. This may increase interference due to the irrelevant unit digit. However, in contrast to two-digit numbers, the neighborhood of the constituents differs between number words and Arabic numbers. The number word corresponding to the *unit* digit is the direct neighbor of the number word corresponding to the hundred digit (i.e., three and seven in above example) whereas the direct neighbor of the hundred digit in Arabic digital notation still is the ten*s* digit (i.e., three and two in above example). If linguistic number word structure influences three-digit number processing, interference due to the unit digit might not be restricted to the neighboring Arabic tens digit but might also extend to the verbally neighboring hundred digit. Consequently, interference due to the unit digit should be more pronounced for German-speaking participants whereas for English-speaking participants it was observed that interference due to the decade digit was more pronounced (Korvorst and Damian, 2008; see also **Figure 1** for an exemplary illustration).

Paralleling the unit-decade compatibility effect for two-digit number comparison, both hundred-decade and hundred-unit compatibility can be defined for three-digit number comparison. A three-digit number pair is hundred-decade compatible when separate comparisons of the hundred and the decade digits lead to the same decisions (e.g., 742_896; hundreds 7 < 8 and decades 4 < 9) and hundred-decade-incompatible when separate comparisons of hundreds and tens lead to opposing decisions (e.g., 362_517, hundreds 3 < 5, but 6 > 1). Analogously, a three-digit number pair is defined as hundred-unit compatible when separate comparisons of hundred and unit digits yield the same decision (e.g., 742_896, hundreds 7 < 8 and units 2 < 6) and hundred-unit-incompatible when these separate comparisons lead to opposing decisions (e.g., 537_692, hundreds 5 < 6, but units 7 > 2). It is important to note that hundred-decade compatibility and hundred-unit compatibility are both attributes of one single number pair, which can be manipulated independently from each other.

So far, there are only few studies investigating language influences in three-digit number comparison tasks. In line with the larger interference due to decade-digits for languages with a non-inverted number word structure, Korvorst and Damian (2008) observed that, for English-speaking adults, the hundred-decade compatibility effect was descriptively more pronounced as compared to the hundred-unit compatibility effect. The authors interpreted this to indicate a left-to-right processing gradient reflecting partially sequential processing. However, it is important to note that in the original stimulus set of Korvorst and Damian (2008) hundred-decade compatibility was confounded with overall distance: overall distance was larger for hundred-decade compatible number pairs. This may have led to an inflation of the hundred-decade compatibility effect and, therefore, questions the proposed interpretation of a left-to-right processing gradient.

The only direct between-language comparison for three-digit numbers was conducted with children in third grade. Klein et al. (2013) observed that only German-speaking children exhibited a reliable hundred-unit compatibility effect, whereas no such effect was found for Italian-speaking children (a language without inversion). Moreover, the hundred-unit compatibility effect was more pronounced as compared to the hundred-decade compatibility effect for German-speaking third and fourth graders (Mann et al., 2012). This corroborates the argument on more pronounced unit interference when units neighboring hundreds verbally due to the inverted structure of German number words. However, the direct comparisons between the language groups was not significant, so that these differential language influences need to be treated with caution.

It is, however, important to note that with any outcome of above study, children’s processing of multi-digit numbers cannot simply be generalized to adults. Children seem to move from a more sequential to a more parallel processing mode for both two-digit (Nuerk et al., 2004; Mann et al., 2011) and three-digit (Mann et al., 2012) numbers. It is well conceivable that language influences children’s more sequential processing of three-digit numbers, but does not influence adult’s more parallel and more automatic (cf. Kallai and Tzelgov, 2012) processing. Therefore, the question remains whether or not a stronger influence of unit interference in a language with inverted number words may only be a transient developmental phenomenon.

The present study set off to investigate inversion-related language specificities in three-digit number processing in German- (a language with inverted) as well as English-speaking (a language with non-inverted number words) adults. Because hundred-decade compatibility was confounded with overall distance in the stimulus set of Korvorst and Damian (2008) we created a new better matched stimulus set avoiding such confounds. Nevertheless, in line with results of Korvorst and Damian (2008), we expect to find reliable effects of hundred-decade as well as hundred-unit compatibility for both language groups indicating that three-digit numbers are processed in a parallel-decomposed manner. Yet, for the English-speaking participants, one would expect no differences in the size of hundred-decade and hundred-unit compatibility effects when the larger hundred-decade compatibility effect were indeed driven by the confounded stimulus set of Korvorst and Damian (2008). On the other hand, we had a specific hypothesis regarding the influence of the inversion property of German number words: for our German-speaking participants the interference due to the verbally first named unit-digit should be more pronounced as compared to the English-speaking participants. This interference should result in a relatively larger hundred-unit compatibility effect as compared to the hundred-decade compatibility effect for the German-speaking sample.

The evaluation of the pattern of compatibility effects for the two language groups will provide first empirical evidence on the question of whether the specific influence of the inverted number word structure on numerical cognition generalizes from 2- to 3-digit number processing. In particular, the proposed differential pattern of compatibility effects would corroborate the notion of inversion-related influences in German to persist into adulthood not only for the processing of two- but also of three-digit numbers.

## Materials and Methods

### Participants

Twenty-five native German speakers (three male, four left-handed) and 28 native English speakers (six male, two left-handed) participated in the study. In each group, one participant had to be excluded due to error rates exceeding 10%. Mean age of the resulting samples was *M* = 23.08 years (SD = 6.28 years) for German- and *M* = 20.11 years (SD = 2.34 years) for English-speaking participants. Participants were recruited via postings at either the University of Tuebingen or the University of York and received course credits or 5€/4£ for compensation. All participants reported normal or corrected to normal vision. The study was approved by the local ethics committee of the University of York.

### Stimuli and Design

In total, the stimulus set consisted of 640 three-digit number pairs. Numbers containing the same digit more than once (i.e., 545 or 555), multiples of hundred (i.e., 200) and/or multiples of ten (i.e., 420) were not included in the stimulus set. For 320 of these number pairs, all corresponding digits differed from each other. For these critical items, the factors hundred-decade compatibility (HDC) and hundred-unit compatibility (HUC; each compatible vs. incompatible), as well as hundred (HD), decade (DD), and unit distance [UD; for all, small (1–3) vs. large (4–8)] were manipulated orthogonally in a 2 × 2 × 2 × 2 × 2 within-subject design. Problem size (the sum of the two numbers of a number pair) was matched for all resulting 32 conditions and overall as well as decade and unit distance was matched for the respective item conditions. Hundred distance could not be held constant for all conditions, as hundred distance is necessarily smaller for hundred-decade-compatible than hundred-decade-incompatible trials when problem size is held constant across conditions [hundred distances: *M_HDC-comp_* = 3.7, *SD_HDC-com_*_p_ = 2.1, *M_HDC-incomp_* = 4.4, *SD_HDC-incomp_* = 2.2; *t*(318) = 3.31, *p* < 0.001]. Descriptive characteristics for these 320 critical number pairs are given in the supplementary material.

Additionally, 320 within-hundred number filler pairs were included. Filler items should prevent participants from focusing on decision-relevant hundred digits only. For half of these filler items, hundred digits were held constant (e.g., 475_421) whereas for the other half hundred and decade digits were identical (e.g., 425_421).

Number pairs were presented above each other in Arabic notation in white against a black background (font: “Arial,” font size: 24 pt, bold) with a viewing distance of ∼50 cm.

### Task and Procedure

In a magnitude comparison task participants had to indicate the larger of two three-digit numbers as fast and accurately as possible. In case the upper number in the display was larger, participants were instructed to press the ‘↑’ key of a standard keyboard with their right index finger. When the lower number was larger, participants had to press the ‘↓’ key with their left index finger. Instructions were given in the respective native language of participants. The two to-be-compared numbers of each pair appeared simultaneously and remained visible until a response key was pressed. Trials were separated by an inter-trial interval of 500 ms. Trial order was randomized separately for each participant. Participants did not receive feedback as to the correctness of their response. Prior to the critical trials, participants performed 10 practice items, which were not part of the stimulus set.

## Results

Only correct responses were considered for analyses [mean error rate was 3.7%, SD = 2.1%; German: 4.0%, SD = 2.3%; English: 3.4%, SD = 1.9%; *t*(49) = 0.86, *p* = 0.392]. All three-digit number pairs with RTs faster than 200 ms were excluded from further analyses. Additionally, all number pairs with RTs deviating more than ±3 standard deviation from the individual participant’s mean RT were excluded. This procedure led to a total loss of 1.4% of the data [German: *M* = 1.4%, SD = 0.4%; English: *M* = 1.4%, SD = 0.7%; *t*(49) = -3.43, *p* = 0.733]. As error rates were very low, analyses focused on RT. Nevertheless, a highly significant positive correlations between error rates and reaction times (German: *r* = 0.788, *p* < 0.001; English: *r* = 0.688, *p* < 0.001) indicated a similar response pattern for errors and RTs disconfirming a speed accuracy trade off. Compatibility and distance effects were evaluated by a 2 × 2 × 2 × 2 × 2 × 2 ANOVA discerning the within-subject factors HDC (compatible vs. incompatible), HUC (compatible vs. incompatible), HD (small vs. large), DD (small vs. large), UD (small vs. large) as well as the between-subject factor language (German vs. English). Mean reaction times and standard deviations for all stimulus categories are presented in **Table 1**. In the following we will first report results relevant for possible language differences in compatibility effects, before describing distance effects as well as further modulations of compatibility effects through distances between respective digit positions.

**Table 1:** Mean and standard deviation for all 32 stimulus categories for the German- and the English-speaking sample.

Hundred distance	Decade distance	Unit distance		Hundred-decade-compatible		Hundred-decade-incompatible	
				Hundred-unit-compatible	Hundred-unit-incompatible	Hundred-unit-compatible	Hundred-unit-incompatible
				
				*M* (SD)	*M* (SD)	*M* (SD)	*M* (SD)
*large*	*large*	*large*	*German*	680 (141)	729 (186)	693 (152)	700 (166)
			*English*	724 (158)	754 (162)	738 (155)	758 (138)
*large*	*large*	*small*	*German*	676 (162)	708 (215)	709 (185)	746 (196)
			*English*	716 (132)	730 (128)	749 (169)	767 (129)
*large*	*small*	*large*	*German*	711 (205)	726 (182)	682 (162)	753 (225)
			*English*	737 (196)	781 (224)	705 (129)	776 (141)
*large*	*small*	*small*	*German*	698 (155)	684 (158)	706 (172)	722 (187)
			*English*	739 (148)	724 (109)	773 (162)	777 (165)
*small*	*large*	*large*	*German*	757 (186)	792 (196)	808 (167)	841 (234)
			*English*	801 (159)	826 (158)	842 (130)	869 (154)
*small*	*large*	*small*	*German*	790 (214)	808 (231)	813 (218)	806 (213)
			*English*	832 (180)	854 (205)	866 (166)	856 (164)
*small*	*small*	*large*	*German*	680 (141)	807 (186)	755 (218)	839 (172)
			*English*	803 (138)	879 (203)	761 (113)	876 (141)
*small*	*small*	*small*	*German*	775 (193)	768 (182)	765 (190)	783 (187)
			*English*	851 (225)	815 (136)	823 (182)	846 (161)

*M, mean; SD, standard deviation*.

### Compatibility Effects and Language Differences

In line with our hypothesis, reliable main effects of HDC and HUC were observed indicating that three-digit numbers were processed in a parallel and decomposed fashion. Hundred-decade compatible number pairs (*M* = 765 ms, SD = 172 ms) were on average responded to 14 ms faster than hundred-decade incompatible number pairs [*M* = 779 ms, SD = 165 ms; *F*(1,49) = 19.65, *p* < 0.001, $\eta_{p}^{2}$ = 0.29]. Additionally, response latencies were on average 27 ms shorter for hundred-unit compatible number pairs (*M* = 759 ms, SD = 165 ms) as compared to hundred-unit incompatible pairs [*M* = 786 ms, SD = 171 ms; *F*(1,49) = 39.96, *p* < 0.001, $\eta_{p}^{2}$ = 0.47].

**FIGURE 2:**
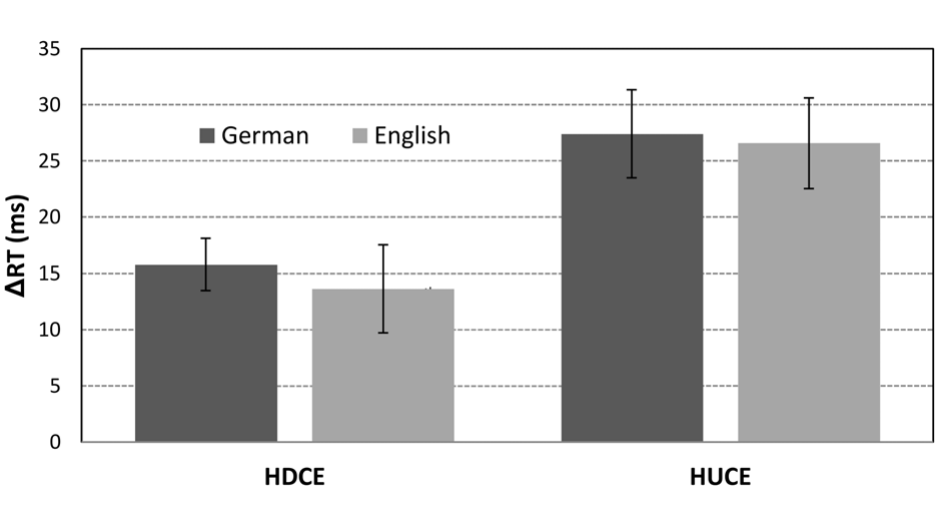
**Effects of hundred-decade (HDCE) and hundred-unit compatibility (HUCE) for German- and English-speaking adults**. Error bars represent standard errors.

In contrast to our hypotheses and in contrast to the compatibility pattern observed by Korvorst and Damian (2008), the main effect of HUC was descriptively larger as compared to the effect of HDC for both language groups (see **Figure 2** for an illustration).

In addition to the main effects of HDC and HUC, the interaction between HDC and HUC was significant [*F*(1,49) = 4.42, *p* = 0.041, $\eta_{p}^{2}$ = 0.08]. In contrast to Korvorst and Damian (2008), this interaction indicated that the effect of HUC is larger for hundred-decade *incompatible* as compared to compatible number pairs (33 and 21 ms, respectively).

In contrast to what we expected, neither the interaction between HDC and language [*F*(1,49) = 0.11, *p* = 0.745] nor that between HUC and language [*F*(1,49) = 0.01, *p* < 0.919] nor the three-way interaction of HDC, HUC and language was significant [*F*(1,49) = 0.10, *p* = 0.756]. Overall reaction times did not differ between groups [German; *M* = 750 ms, SD = 196 ms, English: *M* = 792 ms, SD = 169 ms; *F*(1,49) = 0.81, *p* = 0.372]. Importantly there wasn’t any reliable interaction with the factor language at all. These findings indicate that language did not modulate three-digit number processing. This interpretation is further corroborated by Bayesian analyses. Using the method proposed by Masson (2011), graded evidence for the null hypothesis (given the obtained data) can be calculated (see Masson, 2011, for a detailed description of the method).With respect to the interaction of both HDC and HUC with language, Bayesian analyses revealed that the probability of the null hypotheses (no differences between language groups) was 0.87 and 0.88, respectively. For the three-way interaction of HDC, HUC, and language the probability was 0.87. Applying the criteria suggested by Masson, probabilities above 0.75 can be considered positive evidence for the null hypothesis.

### Distance Effects and Influences of Digit Distances on Compatibility Effects

A significant main effect of hundred distance was found indexing number pairs with a large HD (*M* = 728 ms, SD = 159 ms) to be responded to 88 ms faster than pairs with a small HD [*M* = 816 ms, SD = 176 ms; *F*(1,49) = 534.75, *p* < 0.001, $\eta_{p}^{2}$ = 0.92]. This clearly indicates that number magnitude was processed in the task at hand. Moreover, the effect of DD was marginally significant [*F*(1,49) = 3.33, *p* = 0.074, $\eta_{p}^{2}$ = 0.06]. In line with the results of Korvorst and Damian (2008) this reflected a tendency toward an inverted DD effect: number pairs with a small DD (*M* = 770 ms, SD = 167 ms) tended to be processed faster than pairs with a large DD (*M* = 774 ms, SD = 166 ms). In addition, the interaction of HD and DD was significant [*F*(1,49) = 21.00, *p* < 0.001, $\eta_{p}^{2}$ = 0.30] indicating that the reversed DD effect was only present for small hundred distances but not for large HD (-15 and 7 ms, respectively).

With respect to the HDC effect, a reliable two-way interaction of HDC and DD was observed [*F*(1,49) = 9.30, *p* = 0.004, $\eta_{p}^{2}$ = 0.16]. In line with previous findings on the influence of digit distances on compatibility effects for two-digit numbers (Nuerk et al., 2001) and three-digit numbers (Korvorst and Damian, 2008), this interaction indicated that the HDC effect was more pronounced for larger as compared to smaller DD. Moreover, a reliable interaction of HDC and UD was observed [*F*(1,49) = 4.22, *p* < 0.045, $\eta_{p}^{2}$ = 0.08] indicating that the effect of HDC is larger for large UD when compared to small UD. Additionally, three three-way interactions were observed involving HDC. Firstly, the interaction of HDC and DD was further qualified by the three-way interaction of HDC, DD, and HD [*F*(1,49) = 4.49, *p* = 0.039, $\eta_{p}^{2}$ = 0.08]. Breaking down this three-way interaction into its constituting two-way interactions revealed that the interaction of HDC and DD was significant for small HD [*F*(1,49) = 11.92, *p* = 0.001, $\eta_{p}^{2}$ = 0.19] but not for large HD. Secondly, the interaction of HDC and DD was further qualified by the reliable three-way interaction of HDC, DD, and HUC [*F*(1,49) = 24.16, *p* < 0.001, $\eta_{p}^{2}$ = 0.33]. Breaking down this three-way interaction in two-way interactions of HDC and DD for hundred-unit-compatible and – incompatible number pairs showed that the interaction was significant for hundred-unit compatible number pairs [*F*(1,49) = 28.92, *p* < 0.001, $\eta_{p}^{2}$ = 0.37] but not for hundred-unit incompatible ones [*F*(1,49) = 0.17, *p* = 0.682]. Lastly, the interaction of HDC, UD, and HD was reliable [*F*(1,49) = 19.48, *p* < 0.001, $\eta_{p}^{2}$ = 0.28]. Breaking down this three-way interaction showed that the two-way interaction of HDC and UD was reliable for large HD [*F*(1,49) = 10.49, *p* = 0.002, $\eta_{p}^{2}$ = 0.17] but not for small HD.

For the HUC effect, modulations due to distances between the respective digit positions were observed as well. First, the interaction between HUC and UD was significant [*F*(1,49) = 28.85, *p* < 0.001, $\eta_{p}^{2}$ = 0.37] indicating that the effect of HUC was more pronounced for larger as compared to smaller UD. This two-way interaction was further qualified by two three-way interactions including HD and DD, respectively. Breaking down the three-way interaction of HUC, UD, and HD [*F*(1,49) = 8.89, *p* = 0.004, $\eta_{p}^{2}$ = 0.15] revealed that the interaction of HUC and UD was reliable for both large [*F*(1,49) = 12.11, *p* = 0.001, $\eta_{p}^{2}$ = 0.20] and small HD [*F*(1,49) = 32.32, *p* < 0.001, $\eta_{p}^{2}$ = 0.39]. However, while the HUC effect was larger for large UD for both large and small HD, the difference between the HUC effects for large and small UD was more pronounced for small HD. Breaking down the three-way interaction of HUC, UD, and DD [*F*(1,49) = 26.11, *p* < 0.001, $\eta_{p}^{2}$ = 0.35] showed that the two-way interaction of HUC and UD was reliable for small DD [*F*(1,49) = 42.44, *p* < 0.001, $\eta_{p}^{2}$ = 0.15] but not for large DD.

To further investigate the contribution of inter-digit distances, we ran a regression analysis similar to that conducted by Nuerk et al. (2001) for both language groups separately. Importantly, the results mirrored those of the ANOVA. After checking for collinearity between predictors (e.g., hundred distance was highly correlated with overall distance as well as logarithmic hundred distance), we included the predictors absolute hundred distance, absolute decade distance, absolute unit distance, categorical predictors of HDC and HUC, respectively, continuous predictors of HDC and HUC {e.g., the continuous HDC index for a compatible number pair is positive [732_896; index: +6 (9–3)] while the continuous HDC index for an incompatible number pair is negative [762_851; index: -1 (5–6)]}, and problem size (operationalized as the mean of the two to-be-compared numbers). For both language groups, regression analysis was highly predictive [German: *R* = 0.700, adj. *R^2^* = 0.482, *F*(5,314) = 60.42, *p* < 0.001; English: *R* = 0.718, adj. *R^2^* = 0.507, *F*(5,314) = 66.64, *p* < 0.001] with the same five predictors incorporated in the final model: absolute hundred distance (G: *b* = -0.620; E: *b* = -0.660), HDC categorical (G: *b* = -0.228; E: *b* = -.219), HUC categorical (G: *b* = -0.196; E: *b* = -0.200), HDC continuous (G: *b* = -0.081; E: *b* = -0.105) and problem size (G: *b* = 0.251; E: *b* = 0.190). Thus, the predictors for both languages were identical. Directly contrasting standardized *b*-weights of significant predictors between both languages using the method suggested by Brame et al. (1998) revealed no significant differences for any predictor (all *Z* < 1.1, all *p* > 0.27).

In sum, the results of these regression analyses are in line with previous research: the predictor hundred distance was the strongest predictor of RT indicating that participants indeed performed a magnitude comparison task. In addition, results suggest that even for three-digit numbers, compatibility effects are not simply categorical, but are influenced by the magnitudes of the involved digits. This is reflected by the inclusion of the continuous HDC variable in the final regression model, which did not code compatibility categorically, but defined by magnitude.

## Discussion

The aim of the current study was to investigate whether three-digit number processing in adults is influenced by the specific language property of number word inversion. Although we observed reliable hundred-decade and hundred-unit compatibility effects, these were not modulated by language in the present study. Bayesian analyses substantiated these null effects. In sum, these data argue against a reliable influence of language on three-digit number processing in adults.

This was in contrast to our expectations because such language influences have been found repeatedly for *two-digit numbers* over a variety of tasks and participant groups (e.g., Nuerk et al., 2005; Macizo et al., 2010; Helmreich et al., 2011; Pixner et al., 2011a; Göbel et al., 2013; Imbo et al., 2014). For three-digit number processing, there is first evidence from child data indicating language influences, which were, however, small and not observed consistently (Klein et al., 2013). However, in contrast to this, no language effects were found at all in the present study when evaluating the influence of number word inversion on the pattern of HDC and HUC effects in German- and English-speaking adults using a three-digit number comparison task. This indicates that language influences on multi-digit number processing in children cannot simply be generalized to adults (Nuerk et al., 2004; Mann et al., 2011, 2012).

Nonetheless, comparable and reliable effects of HDC and HUC were observed for both German- and English-speaking adults. This indicated three-digit numbers to be processed in a parallel and decomposed manner in both language groups. Additionally, in both language groups the pattern of compatibility effects differed from the one observed for English-speaking participants previously reported by Korvorst and Damian (2008). In particular, we observed the effect of HUC to be descriptively larger than the effect of HDC contradicting a sequential left-to-right processing gradient in three-digit number processing.

### Lack of Language Differences in Adults

We observed significant HDC and HUC for German and English-speaking adults in ANOVA and regression analyses. In line with previous results for multi-digit numbers (e.g., Nuerk et al., 2001; Ganor-Stern et al., 2007; Macizo et al., 2010; Meyerhoff et al., 2012; Macizo and Herrera, 2013; Moeller et al., 2013) three-digit numbers thus seemed to be processed in a parallel and decomposed manner: the correct decision in number magnitude comparison was not only influenced by the decisive digit (i.e., the hundred digits in the case of between-hundred number pairs) but also by the separate comparisons of decision-irrelevant tens and units. This provides further evidence for the argument that place-value information and the magnitudes of single digits are considered automatically when multi-digit numbers are processed.

Importantly, and in contrast to previous results for two-digit numbers, the present data indicate that three-digit number processing is *not* influenced by number word inversion. The decision-irrelevant tens and units thus exhibited a comparable influence on three-digit number processing for both German- and English-speaking participants. Unlike for tens and units, the position of the hundred digit is not inverted in German number words as compared to its position within the digit string (e.g., *3*84: *drei*hundertvierundachtzig, literally: *three* hundred four and eighty). Considering this, the present pattern of results indicates that interference due to inverted digits does specifically affect the inverted digits but not those next to the inverted ones. In turn, this might mask potential language differences in multi-digit number processing beyond the two-digit number range. Therefore, the observed compatibility effect pattern indicated that language influences observed for two-digit number processing do not generalize to three-digit number processing. Our results thereby indicate that inversion effects seem to be restricted to the digits being inverted (i.e., tens and units) and do not generalize to the verbally neighboring hundreds.

Please note, however, that the samples investigated in this study comprised only 25 and 28 participants, respectively. Therefore, one might speculate that null effects observed in this study might be attributable to power problems and/or type-1 errors associated with small sample sizes. Yet, this seems unlikely for at least two reasons. First, the observed null effects were substantiated by Bayesian analyses indicating them to be reliable. Second, it needs to be considered that for two-digit numbers, influences of inversion on the compatibility effect have been observed with similar sample sizes (e.g., Nuerk et al., 2005). So, even if statistically significant inversion influences on three-digit number processing might be detected with larger sample sizes, this would provide further evidence for our conclusion that these influences are most likely smaller and/or less reliable than for two-digit number processing in adults.

### Differences between Three-Digit Number Processing in Children and Adults

Investigating an adult sample, the present data did not indicate language to influence three-digit number processing. However, when considering previous evidence from children, data indicate language-specific developmental shifts from more sequential to more parallel decomposed processing of multi-digit numbers (e.g., Klein et al., 2013). As previously observed for two-digit numbers, compatibility effects for three-digit numbers seem to become more pronounced with increasing age. However, for non-inverted languages, the effect of *HUC* was present in (English-speaking) adults (this study and Korvorst and Damian, 2008) but not in (Italian-speaking) children (Klein et al., 2013). For the inverted German language, the effect of *HDC* effect was present in adults but not in children of third and fourth grade. Furthermore, for both German-speaking adults (this study) and children (Mann et al., 2012), the effect of *HUC* effect was larger than the hundred-decade compatibility effect. Thus, there are language influences on the developmental shift from more sequential toward more parallel decomposed processing of multi-digit numbers with age and experience.

While the shift in processing patterns can be provoked by changes on the visuo-spatial and/or verbal processing level in non-inverted languages (i.e., English, Italian), in the inverted German language, a shift in processing verbal information is more probable. Because the tens did not interfere with the comparison process in German-speaking elementary school children, it can be assumed that, at least for children up to fourth grade, interference caused by verbal number words is more pronounced as compared to interference caused by the directly neighboring digits in symbolic Arabic number notation. Thus, auditory-verbal neighborhood of spoken number word elements seems to be more important at this point than visuo-spatial neighborhood of written digits. This might be due to children’s tendency to verbalize what they are cognitively engaged in – in the present case three-digit Arabic numbers – more strongly than adults might do. In line with this, research on so-called private speech indicates that children’s use of a more externalized, overt verbal thinking in kindergarten reduces gradually to rather internalized, soundless inner speech over the course of early elementary school (e.g., Kohlberg et al., 1968; Berk, 1992; Winsler and Naglieri, 2003). Although internalizing with age, language-supported processing might still be more pronounced in elementary school children when compared to adults. In turn, visuo-spatial neighborhood seems to become more salient with increasing age, experience, and automaticity in number processing whereas interference due to automatic activation of corresponding number word properties seems to become less salient. Taken together, these findings are in line with the notion of a developmental shift from more *language modulated* sequential processing of three-digit numbers to a more decomposed and parallel processing mode, which gets more independent of language with increasing age and experience.

### Sequential and Parallel Processing and Differential Compatibility Patterns

Combined sequential and parallel processing has been postulated for multi-digit numbers beyond the two digit number range (Korvorst and Damian, 2008; Meyerhoff et al., 2012). For three-digit numbers, Korvorst and Damian (2008) accounted for the larger HDC effect (as compared to the HUC effect) by suggesting a sequential left-to-right processing gradient enhancing the interfering role of the tens. In such sequential processing, tens are assumed to be processed directly following the hundreds and therefore interfere more than the subsequently processed unit digits.

While this explanation is appealing, we were not able to replicate Korvorst and Damian’s (2008) results in this respect. Instead, we observed the HUC effect to be descriptively larger than the HDC effect for both the present English- and German-speaking participants. These results are inconsistent with the assumption of a left-to-right processing gradient. As a consequence, the question why differing compatibility effect patterns were found between the present study and that of Korvorst and Damian (2008) is of theoretical importance for our understanding of multi-digit number processing.

To account for this inconsistency, one should first consider differences in the stimulus set. As already described above, overall distance was larger for hundred-decade-compatible as compared to hundred-decade incompatible number pairs in the original stimulus set used by Korvorst and Damian (2008). Therefore, the HDC effect may have been inflated, because overall numerical distance and HDC were confounded. This confound was eliminated in the present stimulus set, which might have led to the smaller hundred-decade-compatibility effect in the present study. Yet, these contrasting results highlight the importance of matching task, stimuli, and procedures when aiming at evaluating multi-digit number processing.

Nevertheless, differences in stimulus characteristics may not be sufficient to explain that – in the present study – the HUC effect was larger than the HDC effect for both language groups. On a first glance, this seems somewhat counterintuitive since hundred and unit digits are visually and conceptually further apart but caused larger inter-digit-interference. A possible explanation for this finding is the effect of lateral masking. Investigated extensively in reading research, this effect describes the interference letters have on the processing of their neighboring letters. When a target letter is flanked by other letters, parafoveal and peripheral vision decreases and, therefore, the probability of correctly identifying the target letter decreases as well (Wolford and Chambers, 1983; Huckauf et al., 1999). Paralleling letter strings or words, one obvious difference between two-digit and three-digit numbers is that only in a three-digit number the decade digit has two neighbors, whereas the hundred and the unit digit only have one. This means less inhibitory influences for the two lateral digits (e.g., hundreds and units) which might in turn add to a descriptively more pronounced HUC effect.

## Conclusion

Taken together, our data suggest that there are limitations to language influences on multi-digit number processing – at least in adults. We observed no influences of number word inversion on three-digit number magnitude processing. This seems counter intuitive because we have seen an increasing number of papers in recent years showing language influences for a wide variety of numerical task, stimulus sets and participant groups. Therefore, the current data constrain these findings: language influences may not be ubiquitous but seem to be specific to stimulus sets, age groups, and probably tasks. Additionally, our data suggest that perceptual determinants of processing multiple elements deserve attention in multi-digit number processing research. On a practical level, future studies might wish to evaluate the development of language differences with age more systematically. Thereby, possible associations with numerical/arithmetical competencies may be investigated (e.g., Göbel et al., 2013), which, in turn, would allow to better understand the influence of inverted number word systems on children’s numerical development. On a theoretical level, it would be desirable to better the interplay of parallel and sequential processing of in multi-digit numbers. Therefore, future studies may use eye-tracking to evaluate online what is actually going on during the comparison process.

## Conflict of Interest Statement

The authors declare that the research was conducted in the absence of any commercial or financial relationships that could be construed as a potential conflict of interest.
