# Supplementary material for: On the limits of language influences on numerical cognition – no inversion effects in three-digit number magnitude processing in adults
Source: Front Psychol. 2015 Aug 12;6:1216. doi: 10.3389/fpsyg.2015.01216 (PMC4532912; doi:10.3389/fpsyg.2015.01216)
Supplement: Supplementary file 1 [file Table_1.DOCX]

***Supplementary Material***

**On the limits of language influences on numerical cognition – No inversion effects in three-digit number magnitude processing in adults**

**Julia Bahnmueller^1,2^, Korbinian Moeller^1,2,3^, Anne Mann^2^, Hans-Christoph Nuerk^1,2,3^***

^1^KMRC - Knowledge Media Research Center, Tuebingen, Germany

^2^Department of Psychology, Eberhard Karls University, Tuebingen, Germany

^3^LEAD Graduate School, Eberhard Karls University, Tuebingen, Germany

*** Correspondence:** Hans-Christoph Nuerk, Department of Psychology, Eberhard Karls University, Tuebingen, Schleichstr.4, 72076 Tuebingen, Germany.

hc.nuerk@uni-tuebingen.de

**Table A: Mean values of various descriptive variables of the 320 experimental number pairs.**

*(continued overleaf)*
